# Addition of MoodGYM to physical treatments for chronic low back pain: A randomized controlled trial

**DOI:** 10.1186/s12998-019-0277-4

**Published:** 2019-10-25

**Authors:** M. John Petrozzi, Andrew Leaver, Paulo H. Ferreira, Sidney M. Rubinstein, Mairwen K. Jones, Martin G. Mackey

**Affiliations:** 1 Discipline of Physiotherapy, Faculty of Health Sciences, The University of Sydney, Sydney, Australia; 2 Department of Health Sciences, Vrije Universiteit, Amsterdam, The Netherlands; 3 Discipline of Behavioural and Social Sciences in Health, Faculty of Health Sciences, The University of Sydney, Sydney, Australia

**Keywords:** Chronic non-specific LBP, Disability, Self-efficacy, MoodGYM, Secondary psychosocial prevention, Chiropractic

## Abstract

**Background:**

Low back pain (LBP) is prevalent, costly and disabling. A biopsychosocial treatment approach involving physical and cognitive behavioural therapy (CBT) is recommended for those with chronic LBP. It is not known if online psychological coaching tools might have a role in the secondary prevention of LBP related disability. To assess the effectiveness of an internet-delivered psychological program (MoodGYM) in addition to standard physical treatment in patients with chronic non-specific LBP at medium risk of ongoing disability.

**Methods:**

A multisite randomized controlled trial was conducted with 108 participants (aged mean 50.4 ± 13.6 years) with chronic LBP attending one of six private physiotherapy or chiropractic clinics. Disability (Roland Morris Disability Questionnaire) and self-efficacy (Patient Self-Efficacy Questionnaire), were assessed at baseline, post-treatment (8-weeks) with follow-up at six- and twelve-months. Participants were randomized into either the intervention group, MoodGYM plus physical treatments, or the control group which received physical treatments alone.

**Results:**

No statistically significant between group differences were observed for either disability at post-treatment (Effect size (standardised mean difference) 95% CI) RMD − 0.06 (− 0.45,0.31), 6-months RMD 0.01 (− 0.38,0.39) and 12-months − 0.20 (− 0.62,0.17) or self-efficacy at post-treatment PSEQ 0.06 (− 0.31,0.45), 6-months 0.02 (− 0.36,0.41) and 12-months 0.21 (− 0.16,0.63).

**Conclusion:**

There was no additional benefit of an internet-delivered CBT program (MoodGYM) to physical treatments in those with chronic non-specific LBP at medium risk of ongoing disability measured at post-treatment, or at 6 and 12 months.

**Trial registration:**

This trial was prospectively registered with Australian New Zealand Clinical Trials Registry Number (ACTRN) 12615000269538.

## Background

Chronic non-specific low back pain (LBP) is a complex biopsychosocial condition. Evidence suggests underlying psychosocial factors, including poor self-efficacy, depression and pain catastrophising, are possible factors for poor response to physical treatment [1]. A Cochrane review found more effectiveness of biopsychosocial treatments compared to physical treatments for LBP, disability and work status in those with chronic non-specific LBP [2].

Non-specific LBP is the most common form of low back pain, and has an undetermined pathoanatomical cause [3]. Best practice management includes advice about staying active, reassurance that activity will not worsen the problem, exercise [4–7], and a short-course of physical treatments such as manual therapy including spinal manipulative therapy, soft tissue and joint mobilisation [8–12].

Individuals at medium-risk of ongoing disability may have little to no psychosocial issues, compared to those at high-risk, it is argued that some at medium-risk who do not respond to physical treatments may continue to experience ongoing disability that may be due to some psychosocial factors not detected by the STarT Back screening tool (SBST). It is argued therefore that adding a biopsychosocial intervention, such as cognitive behavioural therapy (CBT) to physical treatments, as a secondary prevention measure, for those at medium-risk of ongoing disability may improve outcomes by addressing both physical, and any concurrent psychosocial dimensions of their condition [13]. This combined intervention is endorsed by clinical practice guidelines [10, 12].

CBT interventions improve disability and back pain, as well as self-efficacy and associated depression and anxiety [2]. According to Bandura, the concept of self-efficacy is likened to one’s belief in their ability to complete a task despite the presence of a pain [14]. CBT is usually delivered face-to-face, however, can also be delivered over the internet. Internet-delivered CBT, may be advantageous as it can be provided at no cost and accessed anytime with potentially less societal stigma [15–17]. Internet-delivered CBT has provided immediate and sustained improvements in primary depressive symptoms at three and six months follow-ups [15]. A 2016 systematic review found internet-delivered CBT had small effects on disability, and pain intensity, the baseline levels of psychological distress in these studies was mild to moderate [18].

MoodGYM is a primary and secondary prevention internet-delivered program preventing and managing depressive symptoms in people with troubling but not incapacitating symptoms [19]. It is widely accessible and no cost. MoodGYM consists of five self-help modules (Table 1), exploring thoughts, feelings, stressors and relationships that may contribute to psychosocial distress. MoodGYM has shown small sustained improvements on self-esteem (a similar construct to self-efficacy), effect size 0.16 [20]. MoodGYM has been examined in populations with a chronic condition to manage psychosocial symptoms [21, 22], though has not previously been in back pain populations. MoodGYM was selected in preference to other programs as it has met evidence standards for effectiveness [23] and standards of evidence for public dissemination [24]. This program might be of benefit in people with chronic LBP. Furthermore, other programs available at the time were not as well researched, nor were they as popularly utilised as MoodGYM was with over 1 million registered users [25].

**Table 1:** MoodGYM modules and content

Module	Module content
Module 1	Feelings: Why you feel the way you do
Module 2	Thoughts: Changing the way we think
Module 3	Unwarping: Changing warped thoughts
Module 4	De-stressing: Knowing what makes you upset
Module 5	Relationships: Relationships and how they work out

Therefore, the aim and hypothesis of this study was to determine whether combined MoodGYM and physical treatments was more effective than physical treatments alone for reducing disability and increasing self-efficacy in people at medium-risk of ongoing chronic non-specific LBP.

## Methods

### Design

This study was a single-blinded, multicentred, randomized controlled trial conducted across six chiropractic and physiotherapy clinics in metropolitan New South Wales and Victoria, Australia. The Consolidated Standards of Reporting Trial (CONSORT) guidelines were followed for reporting this randomized trial [26]. The trial protocol was registered prospectively with the Australian New Zealand Clinical Trials Registry Number (ACTRN) 12615000269538 and published [27]. Furthermore, the trial was approved by The University of Sydney Human Research Ethics Committee (protocol number 2014/997). All participants provided written informed consent prior to entering the trial. Recruitment commenced on March 30, 2015 and ended June 2017.

### Participants

Volunteers with chronic LBP were recruited through advertisements in participating practices including, medical and allied health centres in metropolitan Sydney and Geelong, as well on the university trial recruitment website and featured editorials in local newspapers. Respondents were informed about the trial and provided with a Participant Information Statement (PIS) and screened for inclusion by an independent research assistant. Those satisfying the inclusion criteria, and subsequently provided written informed consent were included.

Inclusion criteria: participants over 18 years of age were included if they had non-specific LBP greater than three months duration but had not received manual therapy in the previous three months. The validated risk stratification STarT Back Screening Tool was used to stratify participants at medium risk of ongoing disability [28].

Exclusion criteria: participants were excluded if diagnosed with any of the following: serious spinal pathology (fracture, malignancy, infection, inflammatory disorders, canal stenosis or cauda equina syndrome, spinal cord injury), spinal nerve compromise (determined by the presence of two or more corresponding neurological signs such as dermatomal paresthesia, myotomal weakness, diminished or absent deep tendon reflexes), had undergone spinal surgery in the previous 12 months, were pregnant, had a compensation claim related to their back condition, were unable to independently complete English language questionnaires or unable to independently use a computer.

### Interventions

Participants were randomised into one of two treatment groups: Control Group (1) which received standard care (physical treatments) only, or the Intervention Group (2) which received a combination of standard care (physical treatments) and access to a self-administered program of MoodGYM. Participants were instructed to not reveal their group allocation with their treating practitioner. Protocol was published prospectively [27].

Physical treatments were provided by a registered chiropractor or physiotherapist with more than 5 years’ manual therapy experience. The treatments were provided at no cost to the participants and participants were not precluded from seeking care outside of the trial. The therapists were inducted into the trial and given a regimen of standard physical treatments that were selected pragmatically according to therapist and participant preference. Physical treatments included manual therapy in combination with other modalities such as advice, education and exercise [8, 11, 29]. Manual therapy included spinal manipulation or mobilization and/or soft tissue massage. Advice and education consisted of reassurance and advice about symptom management and encouragement to remain active. Practitioners were instructed to provide key messages that low back pain is mostly benign and self-limiting, principles of activity pacing, along with instruction on safe manual handling, and general postural advice. Participants were also encouraged to remain physically active and avoid excessive bedrest. Exercise therapy included a specific exercise or general conditioning regimen. Specific therapeutic exercise focused on correction of strength, mobility of motor control impairments or general conditioning exercises were prescribed at the discretion of the treating practitioner. Each participant received up to 12 sessions of physical treatment. The frequency and total number of treatments was determined by the clinical judgement and patient response. Treatment was discontinued if the participant experienced either significant improvements in function and/or pain indicating recovery, or a serious or severe adverse response to treatment. The number and type of treatments delivered was recorded by the treating practitioner. Compliance to suggested treatment plan and prescribed exercises was verbally discussed and noted at the time of treatment.

Intervention Group participants received the same physical treatments as the Control Group with the addition of access to the MoodGYM program [30]. We used MoodGYM modules as a secondary prevention tool for teaching people to better respond to troubling emotions or psychological distress. The program presented a combination of written information, real-life examples and quizzes, delivered within the principles of a CBT framework. Module 1 provided information about the felt experience of troubling emotions; module 2 and 3 provided CBT-based information and behavioural exercises that taught participants how to adapt healthier thoughts and behaviours in daily life; module 4 provided information about psychological distress and provided behavioural coping strategies; module 5 presented interpersonal problem solving strategies that could be used to prevent psychological distress in personal relationships. Participants were provided with a MoodGYM user manual briefly outlining the website address and how to create a personal login to the program [31]. No further assistance was provided above and beyond what is already available to public internet users. Participants were instructed to work through one module per week whilst concurrently undertaking their physical treatments. No face-to-face counselling was provided outside of the interaction received with MoodGYM. An online log which could track participant use of MoodGYM was not available to the researchers. However, each participant received a weekly telephone call from an independent research assistant to assess and encourage adherence to the online program. Provision was made for any participants who reported severe distress at time of weekly telephone call as a result of using MoodGYM to be referred to a clinical psychologist.

### Randomisation and blinding

The randomisation process was conducted by an independent research assistant, before the trial commenced. This research assistant was not involved in participant recruitment, treatment delivery, data collection or analysis. A computer-generated random number sequence of 108 treatment assignments resulted in an equal number of 54 assignments between the two groups. Block randomisation was not used. These group allocations (Control or Intervention) were individually sealed in consecutively numbered opaque envelopes by the research assistant. Immediately after participant enrolment, another independent research assistant opened the next available envelope to reveal the participants treatment group allocation. Once allocated, the participant was not able to change groups. The group allocation was only known by the research assistant and the participant. Participants were instructed not to divulge group allocation to their treating practitioner. Furthermore, participants were not fully informed as to the nature of the intervention CBT program at time of recruitment. Namely, participants in the Control Group were not able to purposefully access MoodGYM during the trial as it was not specifically mentioned in the study advertisement, participant information statement or discussed by the treating practitioner or their staff. As the treating practitioner was blinded to group allocation, participants were instructed not to discuss MoodGym with them.

### Data collection and outcome measures

Baseline data collection included demographic and clinical characteristics: gender, age, work status, functional impairment, treatment history and medical history, self-efficacy, disability, pain intensity, pain catastrophizing, stress anxiety and depression and work ability. All outcomes scores were collected again at post-treatment (8 weeks), and at 6- and 12- month follow-up. Participants entered responses directly into an online survey collection database (Survey Monkey©).

### Primary outcomes

Primary outcomes were self-efficacy and low back pain-related disability. Self-efficacy is a persons’ belief and confidence that a physical task can be performed despite sensations of pain [32]. The Pain Self Efficacy Questionnaire (PSEQ) measured self-efficacy, range 0 to 60, where a higher score reflected higher self-efficacy [33].

The Roland Morris Disability Questionnaire (RMD) [34] measured disability, range 0 to 24, where a higher score reflects higher disability [34].

### Secondary outcomes

Secondary outcome were Pain Catastrophizing Scale (PCS) [35, 36], Patient Specific Functional Scale (PSFS) [37], Depression Anxiety and Stress Scale (DASS21) [38], Pain Numerical Rating Scale (PNRS) [39] and Work Ability, using the single item Work Ability Index (WAI) score [31].

All instruments were validated outcomes whose psychometric properties have been outlined previously [27].

### Adverse events outcomes

Adverse events were monitored according to the revised and extended 2003 CONSORT statement for reporting clinical trial data [40]. A serious adverse event from physical treatment was defined as any untoward occurrence resulting in hospitalization, life threatening injury that results in persistent significant disability or incapacity or death [41]. An enquiry for any minor, moderate or severe physical treatment adverse event [42] was made by the practitioner at each treatment session investigating: 1) a new related complaint which was not present at baseline or previous visit, or 2) a worsening of the presenting complaint [43]. Adverse events from physical treatments were measured at each treatment by two questions which the participant self-scored on a 10-point scale from 1(Not at all) to 10 (Extreme increased pain). *Q1. During your treatment, did you experience increased pain or stiffness at the treated area? Q2. During your treatment, did you experience increased pain or stiffness in another treatment-related area?* Adverse events from the psychological intervention (MoodGYM) were monitored by an enquiry at the weekly telephone reminder calls. As part of ethics approval, participants who reported psychological distress from using MoodGYM, were offered the opportunity to contact an experienced clinical psychologist to discuss any issues of psychological distress that may have arisen throughout the entirety of the study. This would be recorded as an adverse event from the psychological intervention.

### Sample size calculations

A total of 108 participants were required to show a statistically significant between-group difference for the primary outcomes (disability and self-efficacy) with a moderate Glass’s delta effect size of 0.60 standard deviations (SD), with a power of 80% (alpha = 0.05) and with a potential 15% drop-out rate. This sample size was also adequate to detect a between-group difference of > 9 points in PSEQ score [44], and a between-group difference of > 4 points in the RMD [44, 45].

### Statistical methods

As this was an exploratory and not a confirmatory trial, we did not use any statistical methods to control for testing multiple outcomes, as recommended by Bender and Lange (2001) [46]. This approach avoided the generation of type II errors [47]. We considered it important to include information of the multiple outcomes measured for descriptive purposes and did not intend them to be used for definitive proof in decision making. Thus, primary and secondary analyses were conducted using the same method of linear mixed models. The effect size was computed as Glass’s delta, that is the mean between group difference/SD of the control group.

Analysis was performed using intention-to-treat. At analysis, all participants were maintained in the group to which they were allocated regardless of non-attendance, withdrawal, or loss to follow-up. The primary analyses used linear mixed models to test for between group differences in PSEQ and RMD at post-treatment (week 8), 6- months and 12- months follow-up. As linear mixed models were used to analyse the data all participants were included in each analysis regardless of any missing data points. This process avoided the need to impute missing data which could have the effect of falsely increasing the power of the study and thus precision in the estimates, and of biasing results towards the null hypothesis. Two tailed significance values of *p* < .05 were considered to indicate statistical significance in between-groups differences. Statistical assumptions were satisfied for this analysis, that is, there were no influential outliers, the residuals were normally distributed, and an appropriate covariance structure was used.

## Results

### Participants

**Fig. 1:**
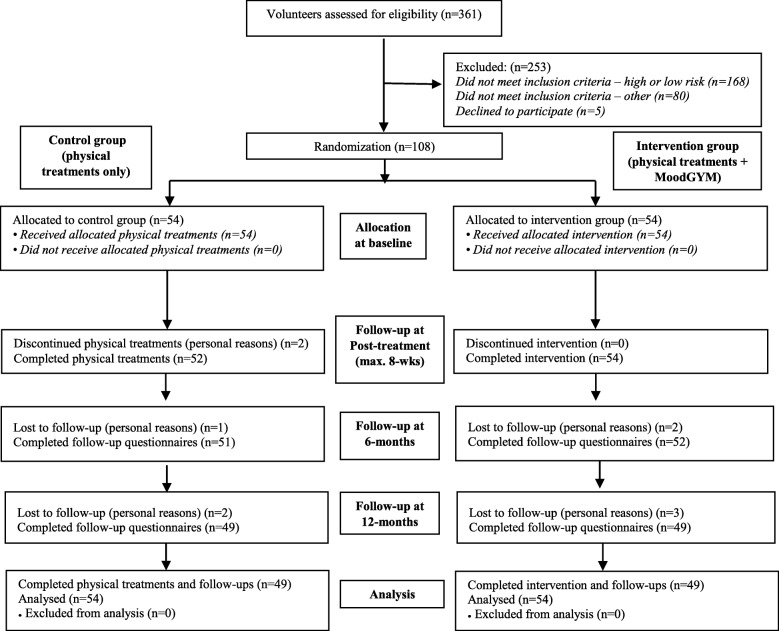
Flow of Participants

Figure 1 details the participant flow through the trial. In total, 361 volunteers were screened for eligibility, of which 253 did not fulfill the inclusion criteria: 168 (66.5%) were classified as at either high-risk or low-risk of ongoing disability according to the STarT Back tool; 80 (31.6%) did not meet other inclusion criteria. Of these 28 had an active workers compensation claim, 12 had recent spinal surgery, 6 were pregnant, 8 had a diagnosed malignancy, 11 had spinal nerve compromise and 15 were unable to independently complete questionnaires in English language. Additionally, 5 volunteers subsequently declined to participate. In total, 108 participants were enrolled. At baseline, there were no significant participant characteristic differences between the two groups (Table 2). In line with the CONSORT statement, we did not statistically test for differences in baseline characteristics. Any differences between the characteristics would have been due to chance and not bias, and baseline testing would merely verify the efficiency of the randomisation process. While it is acknowledged that some authors undertake baseline testing, a systematic evaluation by Peterson et al. [48] argues the practice should be discouraged. Characteristics related to study outcomes were not observed to be largely different between the groups.

**Table 2:** Participant characteristics at baseline by Group (values are mean ± SD for continuous variables and N (%) for dichotomous variables)

	Control group (*n* = 54)	Intervention group (*n* = 54)	Total (*n* = 108)
Participants			
Age (years)	50.6 (14.4)	50.1 (12.8)	50.4 (13.6)
Male	22 (40.7%)	32 (46.3%)	54 (50%)
Female	25 (59.3%)	29 (53.7%)	54 (50%)
BMI	26.7 (4.0)	27 (5.0)	26.8 (4.5)
Pain Intensity			
PNRS (0–10)	4.9 (2.0)	5.1 (1.8)	5.0 (1.9)
Pain Duration			
3–12 months	3 (5.6%)	1 (1.9%)	6 (5.5%)
12 months −2 years	6 (11.1%)	8 (14.8%)	14 (13.0%)
2–5 years	8 (14.8%)	8 (14.8%)	16 (14.8%)
> 5 years	37 (68.5%)	35 (64.8%)	72 (66.7%)
Pain Description			
Always present, level of pain varies	32 (59.3%)	36 (66.7%)	68 (63.0%)
Often present, with pain-free periods < 6 h	14 (25.9%)	10 (18.5%)	24 (22.2%)
Cause of Pain			
Injury at home	3 (5.6%)	5 (9.3%)	8 (7.4%)
Injury at work	8 (14.8%)	8 (14.8%)	16 (14.8)
Motor vehicle accident	4 (7.4%)	2 (3.7%)	6 (5.6%)
No obvious cause	25 (46.3%)	23 (42.6%)	48 (44.4%)
Injury other setting	6 (11.1%)	7 (13.0%)	13 (12.0%)
Functional Status			
RMD (0–24)	9.9 (4.7)	9.9 (4.2)	9.9 (4.4)
PSFS (0–10)	4.1 (1.3)	4.2 (1.5)	4.2 (1.4)
WAI (0–10)	5.6 (2.1)	5.7 (2.1)	5.7 (2.1)
Psychological Status			
PSEQ (0–60)	37.8 (13.0)	40.1 (10.0)	44.5 (12.3)
PCS Total (0–52)	20.9 (12.5)	20.0 (11.3)	20.5 (11.9)
PCS Rumination (0–16)	7.59 (4.3)	7.6 (4.2)	7.6 (4.2)
PCS Magnifying (0–12)	4.2 (3.0)	3.6 (2.8)	3.9 (2.9)
PCS Helplessness (0–24)	9.1 (6.0)	8.9 (5.6)	9.0 (5.8)
DASS21 Total (0–63)	16.8 (12.6)	15.0 (10.1)	15.9 (11.4)
DASS21 Depression (0–21)	5.5 (5.3)	5.3 (4.8)	5.4 (5.0)
DASS21 Anxiety (0–21)	3.6 (3.6)	2.8 (2.9)	3.2 (3.3)
DASS21 Stress (0–21)	7.7 (4.7)	7.0 (3.9)	7.3 (4.3)
Work Status			
Full-time	21 (38.9%)	24 (44.4%)	45 (41.7%)
Part-time	14 (25.9%)	18 (33.3%)	32 (29.6%)
Work hours affected by pain	26 (48.1%)	23 (42.6%)	49 (45.4%)
Work type affected by pain	34 (63%)	33 (61.1%)	67 (62.0%)
Health Status			
Self-reported depression/ anxiety	14 (25.9%)	12 (22.2%)	26 (24.1%)
Osteoarthritis	12 (22.2%)	8 (14.8%)	20 (18.5%)
High blood pressure	10 (18.5%)	4 (7.4%)	14 (13.0%)
Stomach ulcer	2 (3.7%)	4 (7.4%)	6 (5.6%)

*Note*: *BMI* (body mass index), *PNRS* (pain numeric rating scale, *RMD* (roland morris disability questionnaire), *PSFS* (patient specific functional scale), *WAI* (work ability index), *PSEQ* (patient self-efficacy questionnaire), *PCS* (pain catastrophizing scale), *DASS21* (depression anxiety stress 21-item scale)

Participants were middle aged, slightly above normal weight, had moderate levels of back pain, low level of disability, high level of self-efficacy and normal-mild levels of psychological distress.

Employment and health status of participants are also presented in Table 2. Approximately, two-thirds of participants had constant back pain for more than five years. In total, 40% were in full-time employment and approximately half stated that the number of hours worked each week was affected by pain. Furthermore, 62% stated that their type of employment was governed by their experience and expectations of back pain. Forty-four percent did not attribute a specific cause to their back pain. The two most common comorbidities by participants were depression and anxiety (24.1%) and osteoarthritis (18.5%).

### Primary and secondary outcomes

**Fig. 2:**
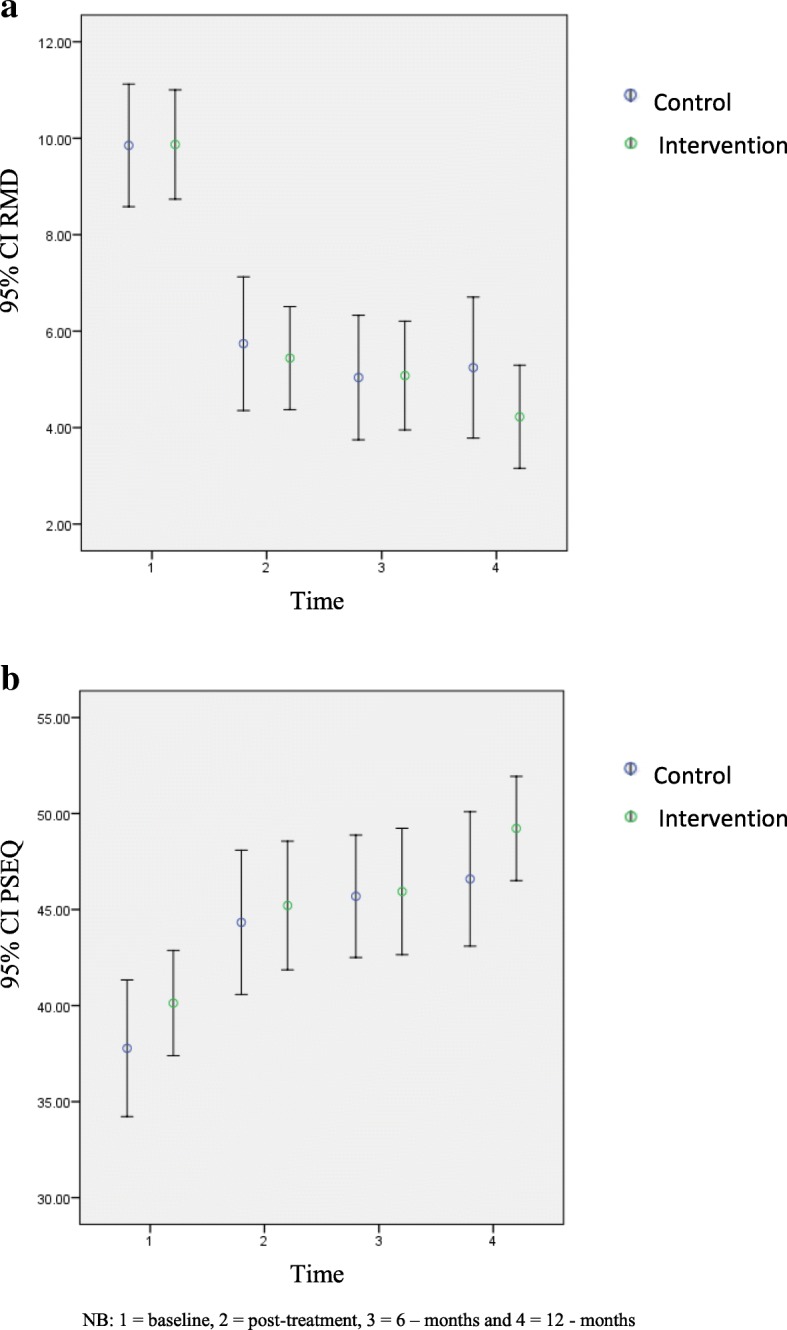
**a** Changes in RMD primary outcomes over time within-groups. **b** Changes in PSEQ primary outcomes over time within-groups

Participants in the control group received mean 7.7 (SD 2.0) physical treatments and those in the intervention group received mean 7.7 (SD 2.4). There was no statistically significant difference between the two groups in either disability (*p* = .70) or self-efficacy (*p* = .52) at any follow-up time points. (Table 3). Between group effect sizes were small to very small and are presented in Table 4. A statistically significant within-group reduction in disability was observed for both groups at post-treatment (*p* < .001) which was maintained at 6 and 12 months. It is not clear whether this change was due to treatment or non-treatment effects such as a regression to the mean. The changes in primary outcomes over time within-groups can be visualised in Fig. 2a and b.

**Table 3:** Mean (SD) and p-value for between group differences using linear mixed models

	Baseline		Post-treatment (max. 8-weeks)		6 months		12 months		Mean differences between groups (0–12 months)
	Mean (SD)		Mean (SD)		Mean (SD)		Mean (SD)		*p* value
Outcome	Control group (*n* = 54)	Intervention group (*n* = 54)	Control group (*n* = 54)	Intervention group (*n* = 52)	Control group (*n* = 52)	Group 2 Intervention (*n* = 51)	Control group (*n* = 49)	Intervention group (*n* = 49)	
Primary									
RMD	9.9 (4.7)	9.9 (4.2)	5.8 (5.1)	5.4 (3.8)	5.0 (4.6)	5.1 (4.0)	5.3 (5.1)	4.2 (3.7)	0.70
PSEQ	37.8 (13.0)	40.1 (10.0)	44.3 (13.8)	45.2 (12.0)	45.7 (11.5)	45.9 (11.7)	47.6 (12.2)	50.2 (9.4)	0.52
Secondary									
Pain and function									
PNRS	4.9 (2.05)	5.1 (1.8)	2.9 (2.0)	2.8 (2.0)	3.2 (2.2)	3.2 (2.2)	4.0 (2.1)	3.0 (2.1)	0.95
WAI	5.6 (2.1)	5.7 (2.1)	6.3 (2.3)	6.5 (2.0)	6.4 (2.3)	6.5 (1.9)	6.2 (2.5)	6.8 (2.2)	0.62
Psychological									
PCS Total	20.9 (12.5)	20.0 (11.3)	14.9 (10.5)	15.5 (11.6)	11.5 (8.8)	13.4 (9.8)	12.8 (10.1)	11.8 (10.0)	0.89
PCS Rumination	7.6 (4.3)	7.6 (4.2)	5.8 (4.1)	5.6 (4.1)	4.6 (3.7)	5.1 (3.7)	5.1 (4.2)	4.5 (3.7)	0.97
PCS Magnification	4.2 (3.0)	3.6 (2.8)	2.8 (2.2)	2.9 (2.6)	2.1 (1.8)	2.4 (2.2)	2.6 (2.2)	2.1 (2.5)	0.69
PCS Helplessness	9.1 (6.0)	8.9 (5.6)	6.4 (4.9)	7.0 (5.7)	4.8 (3.9)	6.0 (4.6)	5.1 (4.4)	5.2 (4.8)	0.61
DASS21 Total	16.8 (12.6)	15.0 (10.1)	13.3 (11.3)	12.2 (9.5)	11.0 (9.0)	10.8 (7.3)	11.2 (9.4)	9.8 (8.1)	0.49
DASS21 Depression	5.5 (5.3)	5.3 (4.8)	4.2 (4.5)	4.2 (4.6)	3.0 (3.0)	3.7 (3.8)	3.5 (3.5)	3.2 (3.5)	0.98
DASS21 Anxiety	3.6 (3.6)	2.8 (2.9)	3.0 (3.3)	2.4 (2.7)	2.6 (3.0)	2.0 (2.1)	2.7 (3.3)	2.1 (2.5)	0.19
DASS21 Stress	7.7 (4.7)	7.0 (3.9)	6.1 (4.3)	5.6 (3.3)	5.4 (3.8)	5.2 (3.1)	5.0 (3.7)	4.4 (3.2)	0.41

Note: The baseline within-group means were calculated from baseline data. The within-group mean estimates for post-treatment 6 and 12 – months were calculated from linear mixed models

**Table 4:** Between-group effect sizes at post-treatment, 6 - months and 12 - months

	Post-treatment		6 - month		12 - month	
Primary Outcomes	Effect Size (95%CI)	Magnitude	Effect Size (95%CI)	Magnitude	Effect Size (95%CI)	Magnitude
RMD	-0.06 (−0.45,0.31)	very small	0.01 (−0.38,0.39)	very small	−0.20 (−0.62,0.17)	small
PSEQ	0.06 (−0.31,0.45)	very small	0.02 (−0.36,0.41)	Very small	0.21 (− 0.16,0.63)	small

Effect Size very small < 0.20, small 0.20, medium 0.50, large 0.80

### Adverse events

No severe or serious adverse events of physical treatment were reported (Table 5). No adverse events were reported by participants using MoodGYM. Some participants reported short-term mild (25.9%) or moderate (8.3%) pain associated with physical treatment. No participants discontinued treatment due to adverse events.

**Table 5:** Adverse events to physical treatments

	Pain at treated area	Pain non-treated area
	%	%
1–2 not at all	63.9	72.2
3–5 mild	25.9	21.3
6 moderate	8.3	4.6
7–10 severe	0.0	0.0

### Treatment adherence

Each participant in the intervention group completed all five MoodGYM modules, confirmed verbally by participants at weekly telephone calls. If a participant stated that they had not started or completed a module by the time of their weekly phone call, they were asked to complete the module within two days. A follow-up phone call was made to ensure that the module had been completed. The participants was instructed to continue with the next module and a weekly telephone call was made the following week.

## Discussion

This study found that a combination of physical treatments and access to the MoodGYM program was not more effective than physical treatments alone for improving disability and self-efficacy in people with chronic non-specific LBP at medium risk of ongoing disability. These results align with previous research which found no clinically significant differences between physical, behavioural/psychologically informed, and combined interventions in those treated for non-specific LBP [49]. That is, a combined treatment was not better than physical treatment alone.

There are several possible explanations why no difference in outcomes between the two treatment groups were found in the present study.

First, selecting participants at the high-risk of ongoing disability may have been a more appropriate target for the current trial. As suggested by Ailliet et al. (2016), psychosocial factors have minimal added value in predicting outcomes in patients presenting to chiropractors with non-specific LBP unless they are in the high-risk group [50]. Furthermore, improvements previously identified in a study of CBT for chronic back pain are predominately related to people with low levels of self-efficacy, high levels of disability and high levels of depression or anxiety, as they generally respond better to CBT [51].

Second, patient self-efficacy of our sample was already relatively high at baseline (mean 44.5, SD 12.3) compared to normative values for people with LBP (mean 25.5, SD 13.4) [33]. Thus, a ceiling effect may have existed at baseline leaving little room for further improvement among participants exposed to the combined intervention. Furthermore, MoodGYM was designed to improve depressive symptoms in non-clinical populations [2, 13, 19, 52]. As self-efficacy is a known mediator in the relationship between chronic pain and depression [53], further research should examine how chiropractic and physiotherapy and interventions can better target self-efficacy with back pain treatments. Furthermore, participants in this study had normal-mild levels of psychological distress and thus may not have been optimal candidates to benefit from this intervention tool. The normative data in the literature for the DASS-21 are as follows: Scores for the depression scale for ‘Normal’ are 0–4, ‘Mild’ are 5–6, ‘Moderate’ are 7–10, ‘Severe’ are 11–13 and ‘Extremely Severe’ are 14+. Scores for the anxiety scale for ‘Normal’ are 0–3, ‘Mild’ are 4–5, ‘Moderate’ are 6–7, ‘Severe’ are 8–9 and ‘Extremely Severe’ are 10+. Scores for the stress scale for ‘Normal’ are 0–7, ‘Mild’ are 8–9, ‘Moderate’ are 10–12, ‘Severe’ are 13–16 and ‘Extremely Severe’ are 17+ [38]. At baseline, the mean score for depression in both the control and intervention groups for the was 5.4, which is within the ‘Mild’ range. The mean score for anxiety in both the control and intervention groups was 3.1, which is within the ‘Normal’ range. The mean score for stress in both the control and intervention groups was 7.3, which is also within the ‘Mild’ range.

Third, the MoodGYM program content may not have been specific enough for people with back pain. This study is the first trial to use MoodGYM in a back pain population. When MoodGYM was used in populations with a specific diagnosis such as Multiple Sclerosis (MS), it was found that participants did not relate to the resources within the modules because it was not specific to them [22]. It is possible that a modification to MoodGYM that includes back pain case examples and resources, may be more meaningful to patients in boosting self-efficacy and improving their funactional disability .

Fourth, it is possible that adherence to MoodGYM was not optimal. Unfortunately, we were not able to ascertain objective data on participants adherence to the program, although participants reported compliance on the weekly telephone reminder calls with the research assistant. Poor adherence has been observed in previous studies using MoodGYM and other internet-delivered CBT programs with adherence levels below 10% [54], and has been noted as a large contributor to poor results for depression outcomes [19, 54–57].

### Strengths and limitations

A strength of this study lies in trialing a biopsychosocial approach in manual therapy practice using a validated, no-cost, readily accessible secondary prevention tool (MoodGYM).

In addition to the discussion points above, there are several major limitations of this trial. The first being the application of MoodGYM to individuals at medium-risk of ongoing disability (individuals with low to no psychological risk factors) [58], as this program was designed specifically for managing depression and anxiety in a non-clinical group, not for improving self-efficacy in an LBP population. Moreover, the trial population had normal-mild levels of psychological distress at baseline. However, at the time of planning this trial, MoodGYM was the most appropriate intervention that had the potential of acting as a secondary prevention psychosocial tool, it had met evidence standards for efficacy and effectiveness criteria set by the Society for Prevention Research (SPR) [23], and had substantially met the standards of evidence for public dissemination [24].

Furthermore, there remains some uncertainty about the use of the SBST in people with chronic low back pain. The tool is reported to perform well for disability in medium risk, however, there remains uncertainty of its overall predictive ability in chronic back pain populations [59].

Further limitations of the study included recruitment methods. Recruitment methods used in the trial may have included two different population groups. That is, participants that responded to media editorials and advertisements may be distinctly different to those directly seeking care at the chiropractic, physiotherapy and GP clinics. Research suggests that people seek care for back pain based on high pain and disability levels, and fear that pain would impact work or life [60]. As the majority of people with chronic LBP do not seek care directly (55.5%) [60] it is possible that those responding to the editorials may have been coping with their pain better than those seeking care, and may have had higher levels of self-efficacy and believed their back pain would not affect their work or life. If recruitment was only offered to those seeking care the level of self-efficacy among participants may have been low at baseline. Participants seeking additional care outside of the trial was permitted in this pragmatic study, however data was not tracked and its potential impact on outcomes was not measured which is acknowledged as a potential limitation of the study findings.

Further research may involve a process evaluation to assess participants experiences of the trial that may inform the development of a back pain specific internet-delivered secondary prevention tool. Furthermore, secondary analysis of data may reveal important psychosocial predictors of treatment outcome which would further inform in-the-field practitioners that treat patients at medium-risk.

Although this study reported negative results, we believe that this trial was important because; (1) it contributes to biopsychosocial research that addresses the secondary prevention of psychological distress in a chronic LBP population; (2) it has tested a well-researched internet-based CBT program content of which could potentially be modified to better assist the chronic LBP population by addressing physical and psychosocial factors.

## Conclusions

There was no additional benefit of an internet-delivered CBT program (MoodGYM) to physical treatments in those with chronic non-specific LBP at medium risk of ongoing disability measured at post-treatment, or at 6 and 12 months. Future trials should investigate the effect of an internet-delivered CBT program in those at high-risk of ongoing disability.

## Data Availability

The datasets generated and/or analysed during the current study are not publicly available, as secondary analysis is currently being undertaken with the intention of being published but are available from the corresponding author on reasonable request.
